# Loss of Leucine Zipper Putative Tumor Suppressor 1 (LZTS1) Expression Contributes to Lymph Node Metastasis of Breast Invasive Micropapillary Carcinoma

**DOI:** 10.1007/s12253-015-9923-x

**Published:** 2015-03-27

**Authors:** Xin-Xin Wang, Bing-Bing Liu, Xiao Wu, Dan Su, Zhengmao Zhu, Li Fu

**Affiliations:** 1 Department of Pathology, Beijing Youan Hospital of Capital Medical University, Beijing, 100069 China; 2 Tianjin Medical University Cancer Institute and Hospital, Key Laboratory of Cancer Prevention and Therapy, Key Laboratory of Breast Cancer Prevention and Therapy, Huan Hu Xi Road, Tianjin, 300060 China; 3 Department of Genetics and Cell Biology, Nankai University College of Life Sciences, Tianjin, 300071 China

**Keywords:** Breast IMPC, *LZTS1*, Gene expression and promoter methylation

## Abstract

Breast invasive micropapillary carcinoma (IMPC) is a rare subtype of breast cancer with a high potential of lymph node metastasis, aggressive clinical behavior, and poor disease-free or overall survival. Expression of leucine zipper putative tumor suppressor 1 (LZTS1) was frequently lost or reduced in breast cancer tissues. This study investigated the expression of LZTS1 protein in breast IMPC tissues using immunohistochemistry. In addition, somatic *LZTS1* mutations and promoter methylation were assessed to determine an association with clinicopathological data from IMPC patients. LZTS1 protein was downregulated in 62 (62 %) of 100 IMPC tissue samples and was significantly associated with lymph node metastasis (*P* < 0.05). A *LZTS1* exon mutation occurred in one of the 53 IMPC cases analyzed, whereas a *LZTS1* intron mutation occurred in 26 of 53 cases. Moreover, *LZTS1* promoter was frequently methylated in IMPC samples and was associated with reduced LZTS1 expression levels in IMPC tissues. These data demonstrated that the loss of LZTS1 expression was associated with lymph node metastasis in patients with IMPC, and *LZTS1* promoter methylation could be responsible for the loss of LZTS1 expression.

## Introduction

Breast cancer is the most frequently diagnosed cancer in women, accounting for 1.4 million new cases and an estimated more than 458,000 cancer-related deaths in 2008 worldwide (Global Cancer Facts & Figures, 2nd Edition, American Cancer Society). Early stage breast cancer typically is not associated with symptoms. Risk factors of breast cancer include female gender, old age, inherited mutations (such as BRCA1 and BRCA2), family history of breast cancer, high breast tissue density, overweight or obese after menopause, use of menopausal hormone therapy (MHT), physical inactivity, and alcohol consumption (Global Cancer Facts & Figures, 2nd Edition, American Cancer Society). These risk factors could activate oncogenes and silence tumor suppressor genes, leading to the development of breast cancer [1]. Moreover, the cause of death for breast cancer patients is usually due to metastasis. Thus, the most important prognostic factor in breast cancer remains the lymph node status, which strongly correlates with disease-free and overall survival of the patients.

Clinically, breast cancer is classified as carcinoma in situ or invasive ductal carcinoma (IDC). Histologically, the vast majority of breast cancer is characterized as IDC. Invasive micropapillary carcinoma (IMPC) is a rare subtype of IDC and was listed by the World Health Organization classification in 2003 [2]. The incidence of IMPC is approximately 4.18 %, and IMPC is frequently mixed with IDC, not otherwise specified (NOS) and a pure IMPC is the micropapillary component ≥90 %. In addition, IMPC is associated with a high invasion potential to lymphatic vessels and metastasis to the axillary lymph nodes [3–5]. However, the underlying mechanisms are poorly understood.

Leucine zipper putative tumor suppressor 1 (LZTS1) was identified by Ishii et al. in 1999 [6]. Ubiquitous expression of LZTS1 protein has been detected in normal tissues; however, it is reduced or lost in different cancer tissues and cells, including gastric, lung, bladder, oral, and kidney cancers [6–13]. Restoration of LZTS1 expression in *LZTS1*-negative cancer cells resulted in the suppression of tumorigenicity, reduced tumor cell growth, and arrested cells at the late S-G_2_/M phase of the cell cycle. Previous studies have shown that LZTS1 expression levels are downregulated in breast cancer tissue and cell lines [14]. Our own data confirmed that LZTS1 expression was reduced or lost in IDC, and reduced LZTS1 expression was associated with lymph node metastasis [15]. Hypermethylation of *LZTS1* CpG islands could be responsible for the reduced expression of LZTS1 in cancer cells [7, 8]. In this study, we detected the expression of LZTS1 protein in IMPC tissues using immunohistochemistry. In addition, we determined whether somatic *LZTS1* mutations and promoter methylation were associated with clinicopathological data from IMPC patients.

## Materials and Methods

### Study Population

In total, 100 pure IMPC breast tissue specimens and 20 normal breast tissues (used as a control) were retrospectively retrieved from The Department of Breast Cancer Pathology and Research Laboratory of the Cancer Hospital of Tianjin Medical University in Tianjin, China. These patients were surgically treated in our hospital between October 2005 and December 2009 and histologically diagnosed as IMPC, independently by two pathologists using the WHO criteria [1]. Since our previous study demonstrated that IMPC was significantly associated with lymph nodes metastasis [16], we grouped them as a single disease entity for this study. All of the patients were women with a mean age of 54 years (range: 29–83 years). None of the patients received preoperative radiation or chemotherapy. Use of human tissue in this study was approved by the Ethics Committee of the Tumor Hospital of Tianjin Medical University, and each patient signed a inform consent.

### Immunohistochemistry

Formalin-fixed and paraffin-embedded tissue blocks were cut into sections. For immunohistochemistry, the tissue sections were first deparaffinized in xylene and rehydrated in a series of graded alcohol. For antigen retrieval, the sections were submerged in 5 mM citrate buffer (pH 6.0), cooked for 1.5 min in a pressure cooker, and then incubated with 3 % H_2_O_2_ in phosphate buffered saline (PBS) at the room temperature for 30 min to inactivate endogenous peroxidase. The sections were further incubated with 10 % normal rabbit serum for 10 min to block non-specific binding of the secondary antibody. Next, the sections were incubated with a polyclonal goat anti-LZTS1 antibody (Santa Cruz Biotechnology, Santa Cruz, CA, USA) at a dilution of 1:75 at 4 °C overnight. The negative control section was incubated with a preimmune serum to replace the antibody. On the next day, the sections were washed with PBS thrice, incubated with a biotinylated anti-goat antibody, and then incubated with streptavidin-biotin-peroxidase (Zhongshan Golden Bridge, Beijing, China). Diaminobenzidine was used as a chromogen substrate to visualize the positive signal. Then, the sections were washed in distilled water and shortly counterstained with hematoxylin and mounted. The sections were finally reviewed under a microscope independently by two pathologists and blindly scored for LZTS1 immunoreactivity into four categories using a previously reported scoring method [9]: +++ or strong (96–100 % positive cells); ++ or moderate (51–95 % positive cells); + or weak (2–50 % positive cells); and—or absent (<2 % positive cells).

### Denaturing High Performance Liquid Chromatography (DHPLC) Analysis

We performed DHPLC analysis to sequence *LZTS1* mutations. We designed different primers to amplify the coding regions of the three exons of *LZTS1* (Table 1). Genomic DNA was extracted form paraffin sections using a DNA tissue kit (Qiagen, Germany) according to the manufacturer’s instructions and then subjected to PCR amplification using AmpliTaq Gold (Applied Biosystems, USA) in a final volume of 25 ml. The system control software (Transgenomic Navigator Software, Transgenomic, USA) gave the running conditions of each amplicon. PCR product was applied to a DNASep column (Transgenomic-Wave 3500A DHPLC system, Transgenomic). All of the PCR products that produced tumor-specific shifts on DHPLC were re-amplified and sequenced with both forward and reverse primers using BigDye sequencing chemistry (Applied Biosystems). The PCR products that resulted in DHPLC shifts with both tumor and germline DNA were also sequenced to identify polymorphisms.

**Table 1:** *LZTS1* primers

Primer location	DNA sequence	Tm (°C)	Size PCR product (bp)
Exon 1	5′-CTCACGGAGCCACGACTG-3′ 5′-CACCCCCATTTTGCTTTC-3′	58	492
Exon 2	5′-CTGGCTCAAGGTCGGCAC-3′ 5′-GCTTGCTACCTCCGTCGG-3′	62	460
	5′-TGGTCACACCCGTGGGACC-3′ 5′-GCTGGCTCTTCTGCGAGGC-3′		389
	5′-GGGCCCGGAGCCCAAAGG-3′ 5′-AGTCGATCCCCCAACATG-3′		406
Exon 3	5′-CCGGGCCACCGCATCCGGAGT-3′ 5′-GTGGGCGGCCCCATGTC-3′	64	435
	5′-GGGCCTGGAGCTGGAGGTCTGT-3′ 5′-GGGGATGCACGGGAGAGC-3′		528

### Bisulfite Treatment, Methylation-Specific PCR (MSP), and DNA Sequencing

We treated genomic DNA with bisulfite as previously described [17]. Briefly, 2 g of genomic DNA was diluted into 50 ml of distilled water and 5.5 ml of 2 M NaOH was added. The mixture was then incubated at 37 °C for 10 min. After that, 30 ml of freshly prepared 10 mM hydroquinone (Sigma, St Louis, MO, USA) and 520 ml of freshly prepared 3 M sodium bisulfite (pH 5.0) (Sigma) were added to each tube. After thorough mixing, mineral oil was added to each tube. The tubes were incubated at 50 °C for 16 h. On the next day, 1 ml of DNA wizard cleanup resin was added (Promega, Madison, WI, USA). The modified genomic DNA was purified and eluted with 50 ml of water following the instructions in the kit. After adding 5.5 ml of 3 M NaOH and incubating at room temperature for 5 min, 1 ml of glycogen (Sigma, St Louis, MO, USA) was added to each sample and then precipitated with 33 ml of 10 M NH_4_Ac (Sigma) and three volumes of ethanol. The DNA samples were then re-suspended in 20 ml distilled water and stored at −20 °C until use.

For the MSP analysis of *LZTS1* promoter CpG methylation, we designed two pairs of primers to cover the CpG enriched region of the *LZTS1* promoter as described previously [15]. PCR amplification was performed in a volume of 25 ml containing 2 ml of bisulfite-treated DNA. The PCR cycles consisted of an initial denaturing at 94 °C for 5 min, followed by 40 cycles of 94 °C for 20 s, 60 °C for 20 s and 72 °C for 30 s, and a final step at 72 °C for 5 min. PCR products were then cloned into a pGEM-T Easy Vector (Promega), and DNA was sequenced by Invitrogen (Carlsbad, CA, USA).

### Statistical Analysis

Statistical analyses were performed using a Mann–Whitney *U* test, Spearman’s Rank-Correlation test, Student’s *t* test and *χ* ^2^ test, respectively. SPSS 15.0 software (SPSS, Chicago, IL, USA) was used to analyze the dat. A 2-sided *P* < 0.05 was considered as statistically significant.

## Results

### Clinicopathological Characteristics of IMPC Patients

Among these 100 Chinese IMPC patients, their mean age was 54 years old. Pathologically, 83 cases (83 %) had a tumor size larger than 2 cm; 90 cases (90 %) showed lymph node metastasis, and 23 (23 %) were lymph node grade III, while 89 cases (89 %) had moderate or poor differentiated tumor and 25 cases (25 %) were triple-negative immune phenotype of tumor.

### LZTS1 Expression in IMPC Tissues and Its Association with Clinicopathological Data from IMPC Patients

**Fig. 1:**
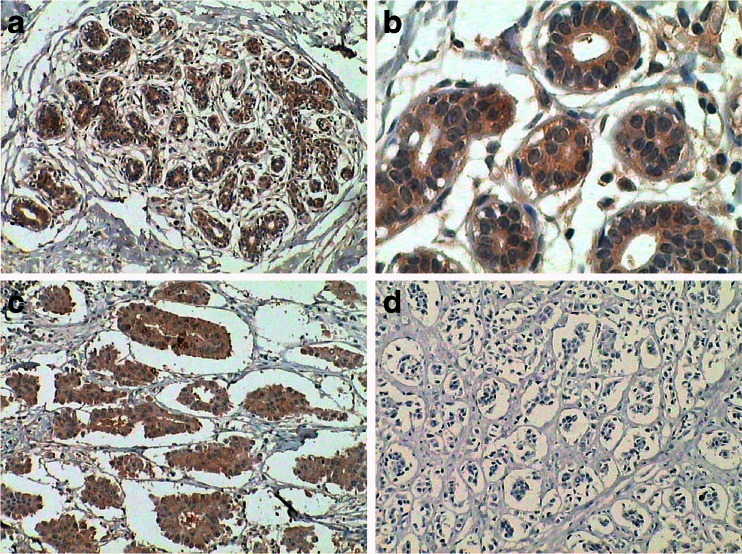
Immunohistochemical detection of LZTS1 expression in normal breast, and IMPC tissues. **a** and **b** Expression of LZTS1 protein in the cytoplasm of normal breast ductal epithelia. **c** Strong expression (+++) of LZTS1 protein in IMPC tissues. **d** Absence of LZTS1 protein in IMPC tissue. **a**, **c**, **d** at ×100 magnification and **b** at ×400 magnification

LZTS1 protein was expressed in normal breast ductal epithelial tissues with a granular localization in the cytoplasm (Fig. 1). In contrast, 62 % (62/100) of the IMPC tissue had LZTS1 protein loss or down-regulation (14 +++, 24 ++, 29 +, and 33 −) and significantly lower than normal tissues (*χ*2 = 25.655, *P* = 0.000; Table 2).

**Table 2:** LZTS1 expression in IMPC and normal breast tissues

Pathologic type	LZTS1 expression		*χ* ^2^	*P* value
	−/+	++/+++		
IMPC	62	38		
Normal	0	20	25.655	0.000

Moreover, we found that among these different LZTS1 expression from strong to absent level, lymph node metastasis rate were 71.4 %, 87.5 %, 96.5 %, 93.5 %, respectively. The expression of LZTS1 protein in IMPC tissue was inversely associated with IMPC lymph node metastasis (rs = −0.210, *P* = 0.036) and lymph node grade (rs = −0.265, *P* = 0.008). However, there was no significant association of LZTS1 expression with patient age, tumor size, histological grade, ER, PR and HER2 expression (Table 3).

**Table 3:** Association of LZTS1 expression with clinicopathological data from IMPC patients

Clinicopathological character	LZTS1 expression				r_s_	*P* value
	(−)	(+)	(++)	(+++)		
Age (mean age = 54)						
<54 years old	18	18	7	4		
≥54 years old	15	11	17	10	0.222	0.026
Tumor size						
<2 cm	4	5	7	1		
≥2 cm	29	24	17	13	−0.062	0.541
Histological grade						
I	2	1	5	3		
II	26	25	16	9		
III	5	3	3	2	−0.143	0.155
Lymph node metastasis						
−	2	1	3	4		
+	31	28	21	10	−0.210	0.036
Lymph node grade						
pN0	2	1	3	4		
pN1	7	10	10	5		
pN2	14	12	6	3		
pN3	10	6	5	2	−0.265	0.008
ER						
−	16	8	8	3		
+	17	21	16	11	0.178	0.077
PR						
−	6	7	7	5		
+	27	22	17	9	−0.137	0.174
HER2						
−/+	27	22	17	11		
++/+++	6	7	7	3	0.067	0.506

### *LZTS1* Somatic Mutation in IMPC Tissues

We extracted DNA from 100 IMPC paraffin-embedded tissues. However, we could only analyze 53 of the DNA samples. In IMPC tissue samples, mutations were localized on exon 3 (C3927G, Leu>Val). Another base change was detected in the intron region (C6544T) in 25/53 of the IMPC samples. These changes were not found in the corresponding normal tissues.

### Methylation of *LZTS1* Promoter in IMPC Tissues

**Fig. 2:**
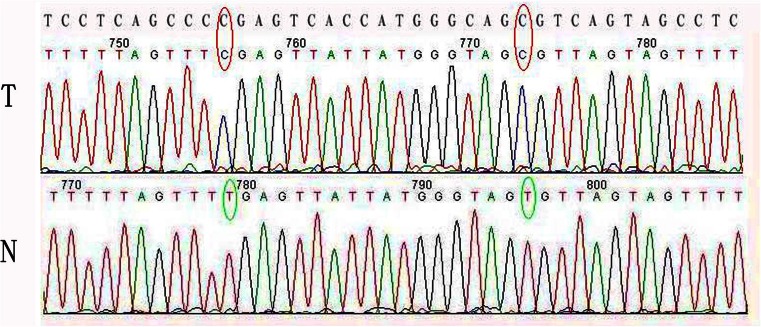
DNA sequencing data of MSP products of a pair of IMPC (*T*) and normal (*N*) tissues. A representative wild-type *LZTS1* sequence is shown at the top. Each of the three cytosines (*C*) in a CpG dinucleotide was converted to thymine (*T*) in the normal tissue, but not in the cancer tissue

**Fig. 3:**
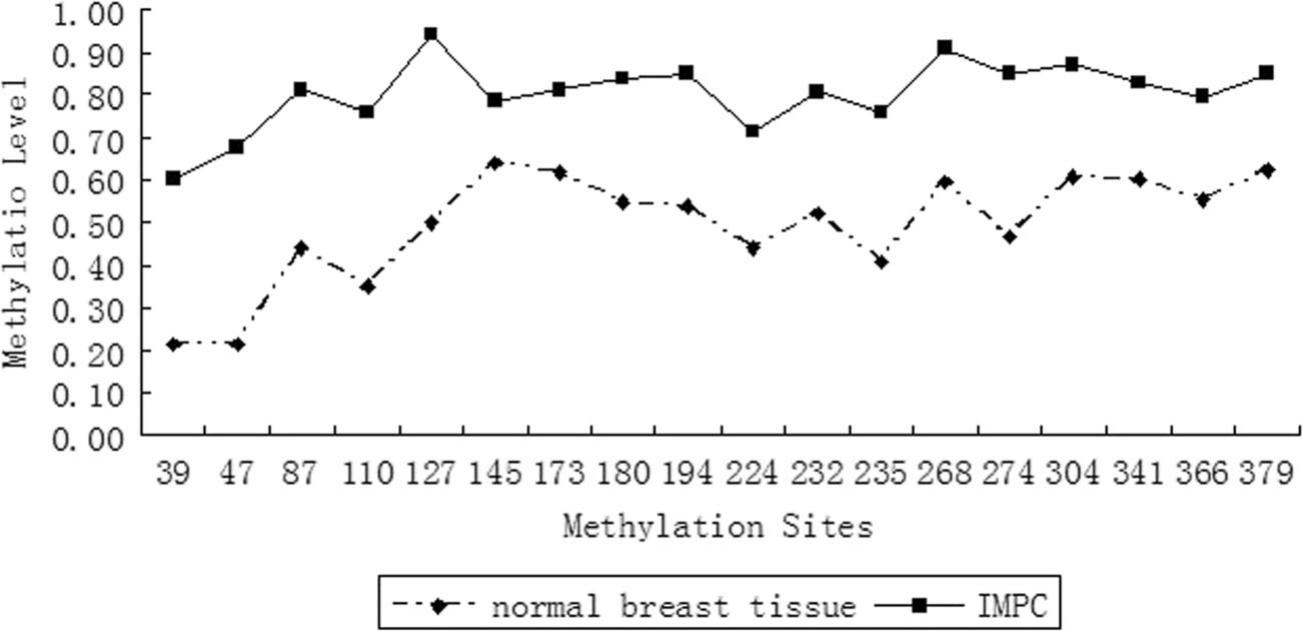
*LZTS1* promoter methylation. *LZTS1* promoter methylation level of each of these 18 CpG dinucleotides in IMPC, and compared to normal breast tissues (*P* < 0.05)

We analyzed *LZTS1* promoter methylation in 18 IMPC samples, and 9 normal breast tissue samples (as a control). After MSP, we cloned PCR products into a pGEM-T Easy Vector and randomly picked ten clones for DNA sequencing from each tumor sample. Figure 2 shows DNA sequence results from normal and cancer tissues. Two CG sites were methylated in the IMPC tumor samples, but not in the normal tissues. The methylation level for each of the 18 CG dinucleotides within the PCR product is shown in Fig. 3. We found that 18/18 (100 %) of the IMPC samples had *LZTS1* promoter methylation. In contrast, 0/9 (0 %) of the normal breast tissue samples had *LZTS1* promoter methylation. Furthermore, Statistical analysis revealed *LZTS1* promoter methylation was associated with the loss of LZTS1 protein expression (Table 4, *P* < 0.05).

**Table 4:** Association between *LZTS1* expression and methylation in IMPC tissues

LZTS1 expression	Methylation			r_s_	*P* value
	Low	Median	High		
+++ / ++	3	0	1		
− / +	1	2	11	−0.607	0.008

## Discussion

In this study, we first detected LZTS1 expression in 100 IMPC tissues. We found that LZTS1 expression was reduced or lost in IMPC, and reduced LZTS1 expression was associated with lymph node metastasis. Many studies have shown that LZTS1 expression is downregulated in different types of human cancers, including cancers of the breast, gastric, lung, bladder, oral cavity, prostate, and kidney [6–13]. In addition, reduced LZTS1 expression was associated with *LZTS1* promoter methylation or mutation [7, 8]. After that, we determined whether somatic *LZTS1* mutations were associated with clinicopathological data from IMPC patients. To date, studies have detected *LZTS1* mutations in different cancers, but they are uncommon. For example, Ishii H et al. [6] analyzed 194 cancer samples, including 72 primary esophageal cancer samples, 18 esophageal cancer cell lines, 24 primary prostate cancer samples, 3 prostate cancer cell lines, 39 primary breast cancer samples, 25 breast cancer cell lines, 8 primary ovarian cancer samples, 4 leukemic cell lines, and 1 cervical cancer cell line. They found two point mutations in two cases of esophageal cancer tissues and one point mutation at codon 501 in the prostate cancer PC3 cell line. Vecchione et al. [8] found a missense mutation in one case of gastric carcinoma that also had *LZTS1* loss. Arnold et al. analyzed *LZTS1* coding regions in 87 primary ovarian adenocarcinoma samples using DHPLC, but only detected a single silent somatic mutation [18]. In this study, we also found one of these 53 IMPC samples had a mutation at exon 3, while 25 of the 53 IMPC samples had a base change in the intron region of *LZTS1*. But *LZTS1* mutation was not the main cause for loss of LZTS1 expression in IMPC.

Gene promoter methylation is a common epigenetic mechanism that transcriptionally inactivates gene expression in different human cancers [19]. Our previous study showed that *LZTS1* promoter methylation was associated with LZTS1 downregulation in IDC [15]. However, *LZTS1* promoter was more frequently methylated in IMPC samples and was associated with reduced levels of LZTS1 expression, indicating that LZTS1 plays a role in promoting IMPC progression at least through the promoter methylation-mediated transcriptional repressor. Therefore, we hypothesized that reactivation of the methylation-silenced gene could restore LZTS1 expression and inhibit IMPC metastasis. The quantitative detection of LZTS1 might be useful for predicting potential of breast cancer metastasis. Indeed, our previous study showed that re-expression of LZTS1 in the highly metastatic MDA-MB-231 cell line resulted in an inhibition of cancer cell proliferation, migration and invasion, and suppression of epithelial-to-mesenchymal transition [20]. However, further studies are needed to confirm this hypothesis.

Our current study is just proof-of-principle and larger sample sizes are needed to confirm our current data. In addition, survival data are needed to determine whether there is an association between LZTS1 expression and overall survival in IMPC patients. Finally, mechanistic studies are needed to investigate the mechanism responsible for *LZTS1* promoter methylation and to investigate the role and functions of LZTS1 protein in IMPC.

## Abbreviations

*LZTS1*: Leucine zipper putative tumor suppressor 1
IMPC: Invasive micropapillary carcinoma
IDC: Invasive ductal carcinoma
DHPLC: Denaturing high performance liquid chromatography
